# Honey Bee Health

**DOI:** 10.3390/vetsci8070127

**Published:** 2021-07-06

**Authors:** Giovanni Cilia, Antonio Nanetti

**Affiliations:** CREA Research Centre for Agriculture and Environment (CREA-AA), Via di Saliceto 80, 40128 Bologna, Italy; antonio.nanetti@crea.gov.it

Honey bee health is a crucial issue that has recently received increased interest from researchers, stakeholders, and citizens.

To explore all possible features of honey bee health, this Special Issue, “Honey Bee health”, aims to explore this topic through a series of research articles focused on different aspects of honey bee health at different levels, including molecular health, microbial health, and population genetic health. All the 21 published articles explore this theme and emphasize the importance of this issue.

Factors associates with honey bee colony losses were reviewed by Hristov et al. [1], and Amiri et al. analysed the interest in honey bee research [2]. Donkersley et al. report a One Health model to reverse honey bee decline [3].

Ribani et al. used environmental DNA (eDNA) to monitor honey bee pathogens and parasites, demonstrating that *V. destructor* is widespread and *L. passim* and *A. apis* with *N. ceranae* occurr frequently together [4].

*V. destructor* infections were modelled, suggesting that colony survival is sensitive to the hive grooming rate and reproductive rate of the mites, which is enhanced in drone-capped cells [5], whereas a new sampling and treatment of this pathogen was found to be a favourable and sustainable method of management [6].

Mendoza et al. found that *V. destructor* resistant honey bees have greater behavioural resistance than susceptible honey bees. At the end of the summer, resistant honey bees had fewer mites and a lower deformed wing virus type A (DWV-A) viral load than susceptible honey bees. Additionally, resistant honey bees were *A. mellifera scutellata* hybrids, whereas susceptible ones were closer to European subspecies [7].

The most promising molecular genetic markers for determining resistance to nosemosis in dark forest bees are microsatellite loci *AC117*, *Ap243,* and *SV185*, which were investigated by Ostroverkhova [8].

Emsen et al. evaluated the seasonality of *N. ceranae* and their relationship with honey bee survivorship, highlighting the highest infection rates, prevalence, and spore viability in the spring and summer, associated with reduced bee populations and food stores in colonies [9].

In the honey bee population in Asia, *Nosema* infection was found in 65% of apiaries by Ostroverkhova et al. Both *N. apis* and *N. ceranae* occur across subarctic and warm summer continental climates, but *N. ceranae* is more predominant in the latter, even if coinfections are predominant (36.3%) [10]. The presence of *N. apis* was also investigated by Naudi et al. in Estonia and Latvia. The results show that *N. apis* is dominant in Estonia (43%) and *N. ceranae* in Latvia (47%) [11].

Porrini et al. studied the effect of compounds commonly used to treat varroosis, evaluating the CHC profiles and EO production on *N. ceranae* infected and non-infected honey bees. The results indicate an absence of alteration in EO or CHC as a response to acaricides ingestion, suggesting that worker honey bees exposed to these highly ubiquitous drugs are hardly differentiated by nest-mates [12].

The efficacy of ApiHerb^®^ and Api-Bioxal^®^ as treatments against *N. ceranae* were investigated using two qPCR methods based on the *16S rRNA* and *Hsp70* genes. Both treatments reduced the abundance of *N. ceranae*, but ApiHerb also decreased the prevalence of infected bees. From the analysis, the qPCR method based on the *Hsp70* gene ensures a higher accuracy for the exact quantification of *N. ceranae* [13].

Dittes et al. discussed the veterinary approach to adult bee examination by analysing the differential diagnosis of the common virus diseases: Acute Bee Paralysis Virus (ABPV)-Kashmir Bee Virus (KBV)-Israeli Acute Paralysis Virus (IAPV)-Complex, Chronic Bee Paralysis Virus (CBPV), and DWV, as well as coinfections such as *Varroa* spp. and *Nosema* spp. [14].

The DWV-A transmission via hive products was investigated in a fully-crossed hoarding cage experiment, estimating the transmission risk by screening commercial products. The results show that DWV-A transmission via hive products is feasible, but the risk of introducing novel viruses and/or strains should be considered [15].

A case report highlights treatment and sanitary measures to save two *A. mellifera carnica* CBPV-infected colonies before the winter [16].

Bullock et al. proposed a silicone wristband as passive samplers in a beehive, developing a novel approach to passively sample honey bee hives. The silicone wristbands provide a simple, affordable, and passive method for sampling the chemical environment of honey bees [17].

Ludvigsen et al. evaluated the honey bee gut mycobiota cluster in different seasons and gut segments. The main finding was that bacteria cluster by gut segments, while fungi cluster by season [18]. Additionally, the administration of veterinary drugs, dietary supplements, and non-protein amino acids affected the ventriculum microbiological profile of *A. mellifera ligustica* [19].

Terenzi et al. reviewed the importance of the sound emitted by the hive. It is used by the bees to communicate within the hive, and its analysis can reveal useful information to understand the colony health status and to detect colony variations [20].

Power et al. found an innovative histological processing technique to analyse healthy drones of *A. mellifera ligustica.* The new approach can detect testes alterations, such as degenerated seminiferous tubules [21].
